# Assessing the digital health readiness questionnaire Japanese version: insights from cardiovascular patients in Japan

**DOI:** 10.1093/ehjdh/ztaf026

**Published:** 2025-05-15

**Authors:** Sanami Ozaki, Toshiki Kaihara, Yoshihiro Akashi

**Affiliations:** Department of Cardiology, St Marianna University School of Medicine, Kawasaki 2168511, Japan; Department of Cardiology, St Marianna University School of Medicine, Kawasaki 2168511, Japan; Department of Cardiology, St Marianna University School of Medicine, Kawasaki 2168511, Japan

## Abstract

**Aims:**

The COVID-19 pandemic has raised patient awareness of their health and highlighted the importance of remote care. Smartphones and wearable devices are now becoming essential for managing cardiovascular disease. However, low digital health readiness among cardiology patients poses a significant challenge to the effective use of these technologies. This study evaluates digital health readiness and learning ability of Japanese cardiology patients using the Digital Health Readiness Questionnaire (DHRQ), while also assessing its reliability and validity.

**Methods and results:**

This multicentre observational study evaluated digital health readiness among patients with cardiovascular risk factors at St. Marianna University Hospital and Kawasaki Municipal Tama Hospital. The DHRQ was employed, and confirmatory factor analysis was conducted to validate the measurement model. A total of 210 questionnaires were distributed, with 208 included in the analysis. Internal consistency, measured by Cronbach’s alpha, exceeded 0.7 across all factors. Model fit was evaluated with standardised root mean square residual = 0.038, root mean square error of approximation = 0.071, comparative fit index = 0.962, and Tucker–Lewis index = 0.955. Age, education, and smartphone/smartwatch ownership significantly predicted higher DHRQ scores. Older age correlated with lower scores (*P* < 0.001), while higher education, smartphone (*P* < 0.001), and smartwatch ownership (*P* = 0.006) correlated with higher scores. Gender and income were not significant.

**Conclusion:**

The DHRQ proved to be valid in Japan, with education level significantly affecting scores. Improved digital health readiness is suggested to enhance patients’ management of health information and communication with healthcare providers, and is expected to be linked to future healthcare systems.

## Introduction

The COVID-19 pandemic accelerated the adoption of digital health tools, including smartphone apps and wearable devices, as alternatives to in-person care. These technologies are increasingly essential in managing cardiovascular disease (CVD), facilitating symptom monitoring, and treatment adherence. However, low digital health literacy, particularly among older adults in Japan, remains a barrier.

Digital health literacy combines ‘digital literacy’ (technological proficiency) and ‘health literacy’ (understanding and using health information). This dual skill set is crucial for individuals to make the most of digital tools that can enhance both personal and public health.^1^ While digital health initiatives can enhance healthcare quality, limited literacy may hinder their benefits.

The Digital Health Readiness Questionnaire (DHRQ), developed in Belgium in 2023,^2^ assesses digital skills, technology use, and learnability, reflecting the evolving digital landscape. Unlike previous tools, it evaluates digital learnability—key for future engagement with digital health. Given the rising CVD burden and its strong association with digital health (e.g. remote pacemaker monitoring), this study targets CVD patients and at-risk individuals.

This study aims to validate the Japanese version of the DHRQ in CVD patients, assessing its reliability and the impact of factors such as digital readiness, device ownership, and education level.

## Methods

### Study design and population

This multicentre observational study was conducted at St. Marianna University Hospital and Kawasaki Municipal Tama Hospital, Japan. It included patients with at least one CVD or risk factor (hypertension, dyslipidaemia, or diabetes) who visited the cardiology departments between 31 October and 10 November 2023. Only fully completed DHRQs were analysed. Patients also provided demographic data, including age, gender, occupation, education, income, marital status, living situation, and device ownership (smartphone, tablet, laptop, smartwatch, or home internet access).

### Digital health readiness questionnaire

Digital Health Readiness Questionnaire responses were recorded on a 5-point Likert scale for agreement (strongly disagree to strongly agree) and frequency (never to daily), scored from 1 to 5, with higher scores indicating greater digital readiness. The total score (15–75) was calculated from the first four factors. A separate ‘digital learnability’ factor (0–25) was also assessed to evaluate potential for digital adaptation. Details of individual DHRQ elements are available in a prior publication.^2^

### Translation and cultural validation

Digital Health Readiness Questionnaire was translated into Japanese by a bilingual researcher (T.K.), then back-translated into English by two independent researchers (T.K. and K.T.). Discrepancies were reviewed, and the revised Japanese version was evaluated by two digital health and cardiac rehabilitation experts. The final version was approved by the original DHRQ drafters (M.S. and M.F.) to ensure content integrity. Given similar smartphone and internet usage rates in Belgium and Japan,^3^ content validity was assumed.

### Statistical analysis

Data analysis was performed using R software (version 4.3.2, R Foundation for Statistical Computing). Categorical variables were expressed as numbers and percentages, continuous variables as means with standard deviations. Internal consistency of DHRQ factors was assessed using Cronbach’s alpha (≥0.7 indicating reliability). Confirmatory factor analysis (CFA) evaluated predefined factors, with model fit assessed using standardised root mean square residual (SRMR < 0.08), root mean square error of approximation (RMSEA < 0.05 ideal, <0.08 acceptable), comparative fit index (CFI > 0.95), and Tucker–Lewis index (TLI > 0.95). Subgroup analysis used unpaired *t*-tests, and multiple regression examined associations between DHRQ scores and covariates (age, gender, education, and income) based on prior research. The CFA sample size was based on prior literature^4^ recommending at least 200, ideally 300, for robust factor analysis and model validation. A two-tailed *P*-value < 0.05 was considered statistically significant.

## Results

A total of 210 questionnaires were distributed; 2 were excluded due to incomplete responses, leaving 208 for analysis. Of the participants, 124 (60%) were male, with a mean age of 68 years (range: 24–96), higher than the original study^2^ (mean: 63 years). Hospital admission reasons included myocardial infarction (18%), angina pectoris (27%), heart failure (25%), diabetes mellitus (26%), and chronic kidney disease (48%). The highest education level was high school (42%) or university (43%). Regarding digital device ownership, 178 (86%) owned a smartphone and 37 (18%) owned a smartwatch. Compared with the original study,^2^ smartphone ownership was higher (86 vs. 81%), while smartwatch ownership was lower (18 vs. 28%). The mean DHRQ score (75-point scale) was 43.6 ± 18.3, and the mean digital learnability score (25-point scale) was 15.0 ± 6.3.

**Figure 1:**
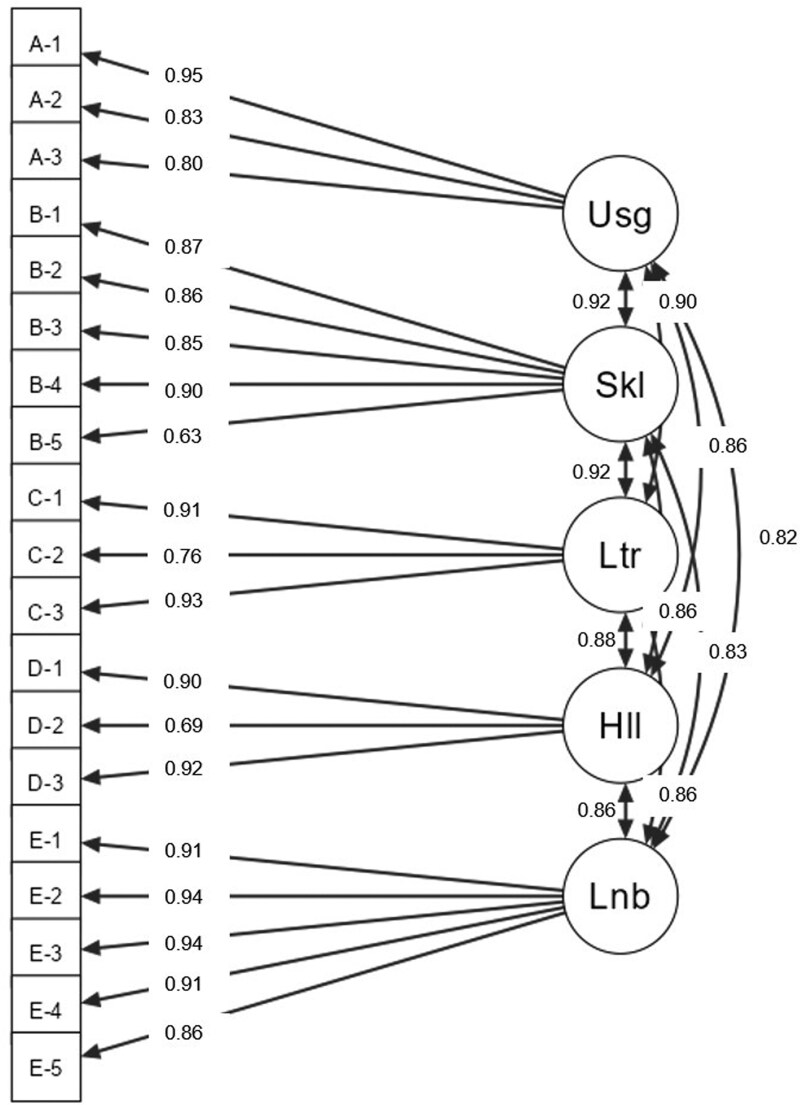
Framework for confirmatory factor analysis without question A-4. Usg, digital usage; Skl, digital skills; Ltr, digital literacy; Hll, digital health literacy; Lnb, digital learnability.

Digital Health Readiness Questionnaire internal consistency was confirmed with Cronbach’s alpha > 0.7 across all five factors. The five-factor structure showed acceptable model fit (SRMR = 0.038, RMSEA = 0.071, CFI = 0.962, and TLI = 0.955). Convergent validity was established (average variance extracted >0.5 across all factors: 0.59–0.83). One variable [A-4: ‘I use wearables (fitness tracker, smartwatch, or other)’] was removed because of the low factor loading (0.33), improving model fit (SRMR = 0.033, RMSEA = 0.067, CFI = 0.969, TLI = 0.963) (*Figure 1*).

Further analysis identified age, education, and smartphone/smartwatch ownership as significant predictors of higher DHRQ scores. Older age correlated with lower scores (*P* < 0.001), while higher education, smartphone ownership (*P* < 0.001), smartwatch ownership (*P* = 0.006), and marital status (*P* = 0.009) correlated with higher scores. Gender and income were not significant (*Table 1*). Subgroup analyses for atrial fibrillation and heart failure showed similar trends in older age, higher education, and smartphone ownership.

**Table 1:** Multiple regression analysis for the total Digital Health Readiness Questionnaire score

Independent variables		Beta (95% CI)	*P*
Age		−0.5 (−0.7–0.4)	**<0.001**
Gender (male)		−0.6 (−4.2–3.0)	0.737
Academic background	Junior high school	—	—
	High school	7.5 (1.5–13.4)	**0**.**014**
	University	15.8 (9.7–22.0)	**<0.001**
	Graduate school	18.6 (9.2–27.9)	**<0.001**
Monthly income (Yen/Euro)	<100 000/641	—	—
	100 000–300 000/641–1923	2.9 (−1.1–7.0)	0.152
	300 000–500 000/1923–3205	1.1 (−4.3–6.4)	0.701
	500 000–700 000/3205–4487	4.0 (−3.3–11.3)	0.284
	700 000–1 000 000/4487–6410	3.1 (−5.9–12.1)	0.499
	>1 000 000/6410	2.7 (−5.4–10.9)	0.507
Marital status (married = 1, unmarried/divorced/widowed = 0)		5.9 (1.5–10.3)	**0.009**
Living situation (living with someone = 1, living alone = 0)		−2.1 (−7.0–2.8)	0.395
Smartphone ownership		14.8 (9.7–20.0)	**<0.001**
Smartwatch ownership		6.1 (1.8–10.4)	**0.006**

Significant *P*-values are in bold. Calculated as 156 Yen per Euro.

## Discussion

This study translated and validated the DHRQ, originally developed in Belgium, for use in Japan. The results confirm its reliability and validity in assessing digital readiness and learnability among Japanese CVD patients. Notably, participants were older and had higher smartphone ownership than in the Belgian study. Subgroup analysis showed that age, digital device ownership, and education significantly influenced DHRQ scores.

The DHRQ demonstrated satisfactory reliability and validity, though the wearable device question had low factor loading, consistent with the original study. Removing this variable improved model fit (*Figure 1*). This may reflect lower wearable device adoption in Japan. Similar findings were reported in the Chinese version.^5^ While Japan has higher smartphone ownership than Belgium, home Wi-Fi and PC ownership are lower (75 vs. 89% and 54 vs. 87%, respectively). Given these trends, digital health strategies in Japan should prioritize smartphones over web-based applications, particularly for device management in cardiology and telerehabilitation. Web-based healthcare applications may require additional support and usability confirmations in Japan.

The *Table 1* highlights education as a key factor, with higher education correlating with greater digital health adoption. Previous research^6^ supports this link, noting that individuals with higher education levels exhibit greater technological literacy and health awareness. It showed significant relationship between DHRQ score and smartphone and smartwatch ownership. Smartphone users likely have better access to healthcare, facilitated by apps and online consultations, improving digital literacy and health engagement.^7,8^

Neither gender nor income significantly affected DHRQ or digital learnability scores. The ‘digital health divide’ suggests education, income, age, and geography impact digital health access. However, in this study, nearly 90% of participants owned a smartphone and had internet access, potentially mitigating income-related disparities. The strong association between education, device ownership, and DHRQ scores highlights the need for early digital health education. Countries like South Korea and Belgium offer free digital literacy programs for older adults. Japan has similar initiatives, such as IT workshops and online community sites for seniors, which should be further promoted.^9^ A systematic review^10^ has shown that digital health literacy interventions—such as electronic health records, educational programs, and telemedicine—enhance patient health management and provider communication. Integrating such interventions into healthcare is crucial. The DHRQ aids in assessing digital literacy and learnability, enabling tailored interventions, such as prioritizing digital health education for high-scoring individuals.

## Limitation

This study has limitations. It was conducted in two urban hospitals, limiting generalizability to rural areas. The brief nature of the DHRQ may not fully capture respondents’ experiences, and biases such as social desirability and self-selection may be present. Cultural and social factors were not extensively examined, but as a validation study, the primary aim was to assess the DHRQ’s applicability in Japan. Due to low wearable device use, one question was removed, improving suitability for the Japanese population.

## Data Availability

Data available on request.
